# Developmental Alterations of Colonic microRNA Profiles Imply Potential Biological Functions in Kid Goats

**DOI:** 10.3390/ani12121533

**Published:** 2022-06-14

**Authors:** Qiongxian Yan, Lina Tian, Wenxun Chen, Jinhe Kang, Shaoxun Tang, Zhiliang Tan

**Affiliations:** 1 CAS Key Laboratory of Agro-Ecological Processes in Subtropical Region, Hunan Provincial Key Laboratory of Animal Nutritional Physiology and Metabolic Process, Institute of Subtropical Agriculture, The Chinese Academy of Sciences, Changsha 410125, China; yanqx14@isa.ac.cn (Q.Y.); tianlina18@mails.ucas.ac.cn (L.T.); chenwenxun@189.cn (W.C.); kangjh@isa.ac.cn (J.K.); zltan@isa.ac.cn (Z.T.); 2 Hunan Co-Innovation Center of Animal Production Safety, Changsha 410128, China

**Keywords:** goat, colon development, highly expressed microRNA, dynamic microRNA

## Abstract

**Simple Summary:**

The colon plays a crucial role in the fermentation and utilization of cell wall carbohydrates, cellulose, and hemicellulose in ruminants. MicroRNAs have been reported to be involved in regulating the gastrointestinal development and maintaining intestinal homeostasis of mammals. In this study, we first reveal the microRNA expression profiles in the colon of kid goats. Meanwhile, we found that targeted genes of DEmiRNAs were highly enriched for the prevention of microbial invasion via the Erbb−MAPK network while targeted genes of HEmiRNAs contributed to the permeable barrier maintenance and surveillance of cell damage. Additionally, more than half of the microRNAs showed a developmentally dynamic expression in the colon of kid goats. This study disclosed microRNA-based biological functions in the regulation of colon development in ruminants.

**Abstract:**

The colon is a crucial digestive organ of the hind gut in ruminants. The bacterial diversity and mucosal immune maturation in this region are related to age. However, whether the microRNA expression in the colon of goats is affected by age is still unclear. In the current study, we analyzed the transcriptomes of colon microRNAs during preweaning (Day 10 and Day 25) and postweaning (Day 31). A total of 1572 microRNAs were identified in the colon tissues. Of these, 39 differentially expressed microRNAs (DEmiRNAs) and 88 highly expressed microRNAs (HEmiRNAs) were screened. The target genes regulated by the DEmiRNAs and HEmiRNAs were commonly enriched in the MAPK signaling pathway, Wnt signaling pathway, Hippo signaling pathway, cell adhesion molecules, focal adhesion, and adherens junction. Remarkably, the targeted genes of the DEmiRNAs were highly enriched for the prevention of microbial invasion via the Erbb−MAPK network while the targeted genes of HEmiRNAs contributed to the permeable barrier maintenance and cell damage surveillance. Additionally, there were eight different expression profiles of 87 dynamic miRNAs, in which approximately half of them were affected by age. Taken together, our study reveals the different roles of DEmiRNAs, HEmiRNAs, and dynamic microRNAs in the development of the colon and gives new insights into the regulatory mechanism of colon development in goats.

## 1. Introduction

Compared with the rumen’s histological structure that has a multilayered squamous epithelium [1,2], the large intestine is covered by a single layer of epithelial cells, where microbial fermentation frequently occurs in ruminants [3]. Specifically, the cecum and proximal colon of ruminants are the second largest sites of fermentation and play an important role in the fermentation and utilization of cell wall carbohydrates, cellulose, and hemicellulose [4]. Additionally, the colonic epithelium is a physical and immunological barrier and inhabited by mucosa-associated bacteria [5]. The bacterial diversity and mucosal immune maturation in this region are related to age in goats [6]. Cell types in the colon include absorptive epithelial cells, mucosal secretory cells, immune cells, and enteroendocrine cells. These special cells contribute to the secretion of protective substrates (such as mucus and antimicrobial peptide) and enzymes into lumen, promoting nutrient absorption and secreting hormones [7,8].

For newborn ruminants, due to the undeveloped rumen, reticulum, and omasum, with their small size and thin wall, milk digestion occurs mainly by enzymes in the abomasum. As the nutrient source of the body gradually changes from liquid feed (colostrum, milk, or milk replacer) to solid feed (starter and forage), the morphology of the digestive tract also undergoes a series of changes. The weight proportions of the rumen and reticulum increase while the weight proportion of the hindgut decreases, and the microvilli become shorter, which morphologically prepares for the transformation of nutrient absorption and utilization [9]. As kid goats age, the phylum Proteobacteria in the large intestine is displaced by Firmicutes, and the microbiota displays taxonomic dynamics with increased *Ruminococcaceae UCG 005*, unclassified *Lachnospiraceae*, *Barnesiella* and *Blautia*, which displays distinct temporal and spatial specificity compared to the foregut [10]. Biological active components from bovine and human milk, such as oligosaccharides, exosomes, and their cargos, contribute to the recovery of damaged colonic mucosa and affect microbial communities in the murine gut [11,12,13]. Solid starter feeding or concentrate supplementation alters colonic mucosal bacterial communities and modulates mucosal immune homeostasis in newborn lambs [6,14].

Recent studies have shown the crucial roles of microRNAs in the rumen development of bovine [15,16] and goats [17] as well as the maintenance of intestinal homeostasis in sheep [4]. Accordingly, microRNA expression profiles in the rumen, small intestine (duodenum and jejunum [18]), and large intestine (cecum and colon) of cattle and sheep have been revealed. The research has also shown that several microRNAs are only expressed in particular intestinal segments, suggesting their roles might be limited to the local microenvironment. Additionally, species differences in the regulation of gene expression by microRNAs occur primarily at the level of expression and processing [19]. In goats, only the rumen microRNA expression profile has been identified [17]. Therefore, exploring the microRNA expression profiles in the large intestine of goats by age is helpful to understand the potential regulatory mechanism of functional microRNA in the hindgut development.

The current study aimed to (1) investigate regional miRNA expression profiles of colon tissues collected from the preweaning stage to the postweaning stage and (2) analyze whether differentially expressed microRNAs (DEmiRNAs) and highly expressed microRNAs (HEmiRNAs) are varied with age. This study reveals microRNAs-based biological functions in the regulation of colon development in ruminants.

## 2. Materials and Methods

### 2.1. Experimental Animals and Colon Tissue Collection

Fifteen newborn and male Liuyang black goats with similar body weights were selected as experimental animals. Goats were breastfed before the age of 28 days and fed *Alfalfa* hay and a powdery concentrate after day 28. Goats were reared according to the feeding standard (NY/T816-2004). The colon proximal segments were collected at three different periods (five samples at each timepoint, 10, 25, and 31 days after birth, represented as D10, D25, and D31, respectively), frozen in liquid nitrogen immediately after dissection, and then stored at −80 °C. The D10 and D25 timepoints represented the preweaning and suckling period while D31 represented the postweaning and solid food feeding stage. The animal study was reviewed and approved by the Animal Care Committee according to the Animal Care and the Use Guidelines of the Institute of Subtropical Agriculture, Chinese Academy of Sciences, Changsha, China (No. ISA-201911).

### 2.2. RNA Isolation, Library Construction and Sequencing

The total RNA from the colon tissues was extracted using a TRK-1002 total RNA purification kit (LC Sciences). The total RNA quantity and integrity were assessed by using a Bioanalyzer 2100 with an RIN number of more than 7.0 and an RNA 6000 Nano LabChip kit (Aglient, Santa Clara, CA, USA) with a 28S and 18S ratio between 1.8 and 2.2 [20]. Approximately 1 ug of total RNA from each sample was used for library preparation. Then, fifteen small libraries (five from each stage) were used for the Illumina/Solexa deep sequencing. Ligation of the total short RNAs (~18–26 nt in length), the reverse transcription, and PCR were performed according to previous work [21]. Finally, the amplification products were used directly for cluster generation and submitted to LC-Bio (Hangzhou, China) for the single-end sequencing on an Illumina Hiseq2500.

### 2.3. miRNA Data Analyses

Firstly, the adapter dimers, junk, low complexity, common RNA families, and repeats of raw reads were removed by the ACGT101-miR program (version 4.2, LC Sciences, Houston, TX, USA) [22,23,24]. Then, the mapping of unique sequences with a length of 18~26 nucleotides to *Capra hircus* (CHIR_2.0) genomics in the miRBase 21.0 database [25] was performed. The identification criterion of known miRNAs or novel 5p- or 3p-derived miRNA candidates was referred to in our previous literature [26]. In order to correctly copy numbers of miRNAs among fifteen different samples, a modified global normalization with basic assumptions and procedures [27] was adopted. The number of reads, which was higher than the average copy of the identified miRNA data set, was defined as HEmiRNAs. All the identified miRNAs based on the normalized deep-sequencing counts (NDSC) were analyzed using the one-way ANOVA, and the caprine and novel miRNAs with *p* < 0.05 were defined as dynamic miRNAs. The trend analysis of the dynamic miRNA was analyzed by a Short Time-series Expression Miner (Version: 1.3.11) [28,29].

MicroRNAs were regarded as differentially expressed based on normalized deep-sequencing levels (with the exclusion of 10 reads of the exon model per million mapped reads). The ANOVA test was used to analyze differences between the three groups. The *p*-value was calculated by using the *p*-value calculation model based on normal distribution. Differentially expressed miRNAs (DEmiRNAs) based on NDSC were analyzed by employing the DEseq R package [30]. The significance threshold was set to a *p*-value less than 0.05 and the absolute value of log2 (fold change) ≥1. The Venn diagram and heatmap of DEmiRNAs were performed by the OmicStudio tools (https://www.omicstudio.cn accessed on 15 May 2021 and 17 May 2021, respectively) using the Venn diagram (Version: 1.6.20) and the pheatmap package (Version: 1.0.12). To predict the genes targeted by the most abundant miRNAs, two computational target prediction algorithms (TargetScan 5.0 [31,32,33] (http://www.targetscan.org/ accessed on 20 May 2021) and Miranda 3.3a [34] (http://www.miranda.org/ accessed on 21 May 2021)) were used to identify miRNA binding sites. Briefly, target genes predicted by the two software programs were screened according to the scoring criteria of each software. Target genes with a context score percentile less than 50 were removed from the TargetScan algorithm. Target genes with a maximum free energy greater than −10 were removed from the miRanda algorithm. Finally, the intersection of the two software programs was taken as the final target gene cluster. The Kyoto Encyclopedia of Genes and Genomes (KEGG) pathway (http://www.genome.jp/kegg/ accessed on 23 May 2021) of the DEmiRNAs and the most abundant miRNAs were also annotated. Significant enrichment was identified at the *p*-value of the Fisher’s Exact Test < 0.05. The bubble diagram of the KEGG pathway enrichment was drawn using the ggplot2 package (R package) [35,36,37].

### 2.4. qPCR Validation of miRNA

To validate the miRNA-sequencing data, six miRNAs randomly selected from the identified DEmiRNAs and certain target genes were examined by a qPCR. The total miRNA was extracted from colon tissues by the EasyPure miRNA kit (ER601-01, Transgen Biotech, Beijing, China) and then transcribed to cDNA using the TransScript miRNA First-Strand cDNA Synthesis SuperMix (AT351-01, Transgen Biotech, Beijing, China). The reverse transcription primer for the miRNA was GATCGCCCTTCTACGTCGTATCGTCATCTGACCGTTATCGCTGCACGTTTTTTTTTTTTTTTTTTTT. The universal reverse primer was GATCGCCCTTCTACGTCGTAT (Tm = 58 °C). The primer sequences for qPCR are listed in the Supplementary Table S1. The qPCR was carried out using the PerfectStartTM Green qPCR SuperMix (AQ601, Transgen Biotech, Beijing, China) on a LightCycler 480II Real-time PCR Detection System (Roche, Germany). Briefly, a volume of 10 μL reactive system included 5 µL 2 × PerfectStartTM Green qPCR SuperMix, 0.2 µL (400 nM) forward primer, 0.2 µL (400 nM) reverse primer, 3.8 µL RNase-Free ddH_2_O, and 80 ng cDNA. The qPCR reaction conditions were as follows: 94.0 °C for 30 s and 40 cycles at 94.0 °C for 5 s, 60 °C for 30 s, and 72 °C for 10 s. U6 was used as a reference gene. The expression level of miRNA was calculated with the 2^−∆∆CT^ method [38]. All samples were run in triplicate. 

### 2.5. Statistical Analyses

Quantitative data from the qPCR was analyzed by a one-way analysis of variance using SPSS 19.0 (SPSS Inc., Chicago, IL, USA). The statistical model included the age of the goat as a fixed effect. Statistical significance was declared at *p* < 0.05. *p*-values between 0.05 and 0.10 were considered trending towards significance.

## 3. Results

### 3.1. miRNAs Expression Profile in the Colon Tissues

In the present study, an average of 9.07 million single-end clean reads were obtained (Supplementary Table S2). A total of 1572 microRNAs were identified in the colon tissues, including 393 known microRNAs, 248 conservative microRNAs, and 296 novel microRNAs. Eighty-eight microRNAs were, coincidentally, highly expressed in the colon tissues at the three developmental periods (Supplementary Table S3).

To compare the consistent changes in microRNA expression among three time-points, 86 dynamic miRNAs were screened (Supplementary Table S4). There were eight different expression profiles in total, in which three profiles were affected by the age (*p* < 0.05). Notably, 12 miRNAs decreased by the age (*p* = 0.024). Twenty-four miRNAs first decreased at D25 and then remained unchanged at D31 (*p* = 0.023). Additionally, 12 miRNAs, including chi-miR-29a, chi-miR-29c, chi-miR-30F, chi-let-7b, chi-miR-92a, etc., increased with the age (*p* = 0.024). Other dynamic miRNAs were not altered by the age (*p* > 0.05).

**Figure 1:**
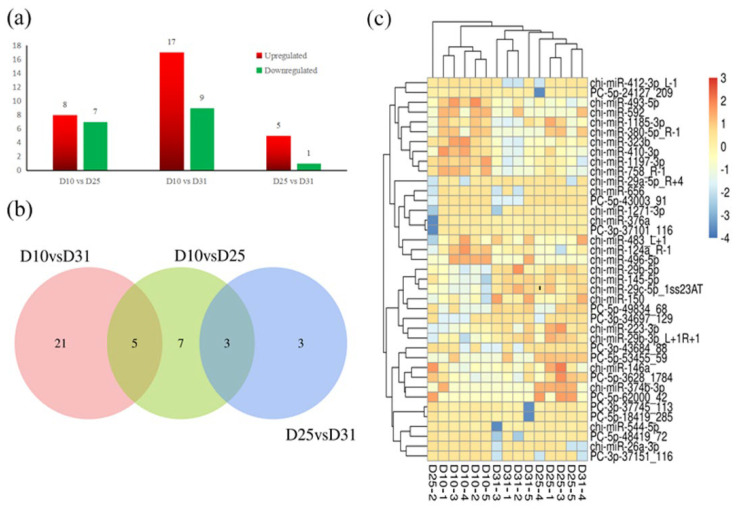
Characterization of differentially expressed miRNAs (DEmiRNAs) in the colon of goats at the preregurgitation stage with a fold change > 2.0 and *p* < 0.05. (**a**) Numbers of DEmiRNAs in the goat colon at D10, D25, and D30. (**b**) Common and unique DEmiRNAs in different pairwise comparisons. (**c**) Hierarchical clustering analysis of DEmiRNAs.

A small number of DEmiRNAs were observed between D10 and D25 (eight upregulated and seven downregulated) or between D25 and D31 (five upregulated and one downregulated, Supplementary Table S4). Fortunately, compared to D10, 17 upregulated microRNAs and 9 downregulated microRNAs were observed at D31 (Figure 1a). The Venn diagram (Figure 1b) shows the DEmiRNAs in each comparison of two different developmental stages. The hierarchical cluster of these 39 DEmiRNAs showed a consistent expression tendency for each stage (Figure 1c).

### 3.2. Pathway Analyses of the DEmiRNAs and HEmiRNAs

**Figure 2:**
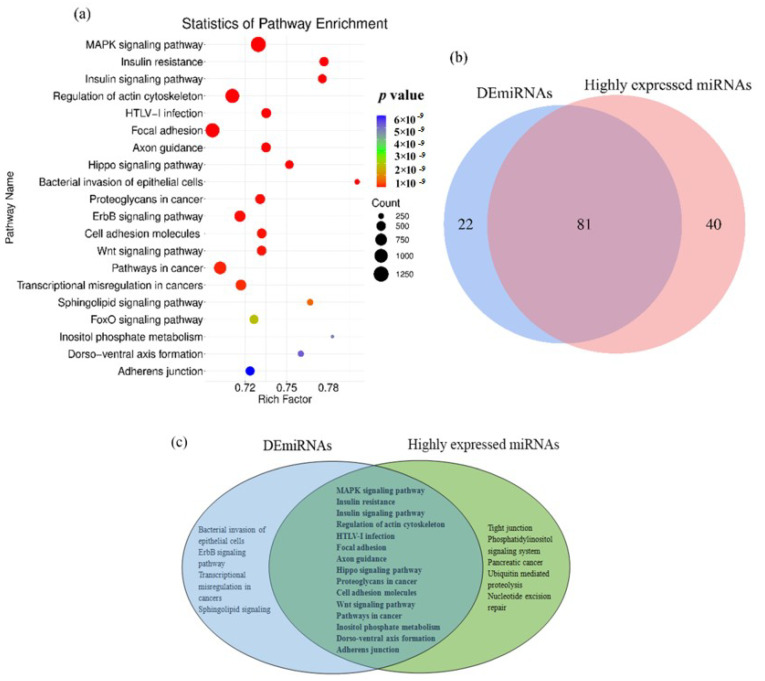
KEGG pathways of the DEmiRNAs and highly expressed microRNAs in the colon of goats at the preregurgitation stage. (**a**) Top 20 enriched KEGG pathways of the DEmiRNAs. (**b**) Venn diagram of the enriched pathways of DEmiRNAs and highly expressed microRNAs. (**c**) The overlap pathways between the DEmiRNAs and the highly expressed microRNAs.

**Figure 3:**
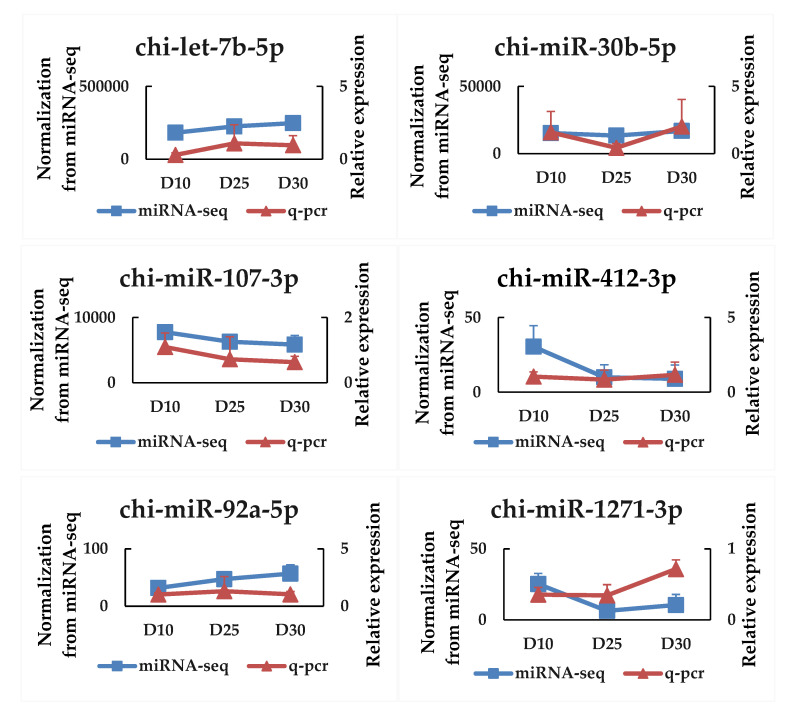
Validation of six selected DEmiRNAs by qPCR.

The biological pathways of the DEmiRNA and HEmiRNA target genes involved in the colon development were revealed by the KEGG analysis. In total, 103 and 121 signal pathways were considered significantly enriched (*p* < 0.05) based on all the DEmiRNA and HEmiRNA highly expressed microRNAs (Supplementary Tables S6 and S7), respectively. Consequently, 81 signal pathways were overlapped between DEmiRNAs and HEmiRNAs, including the MAPK signaling pathway, Wnt signaling pathway, Hippo signaling pathway, cell adhesion molecules, focal adhesion, and adherens junction (Figure 2). To verify the expression pattern of DEmiRNAs, six microRNAs were randomly selected and tested by qPCR. Accordingly, the results were consistent with the expression tendency of the microRNA-sequencing data (Figure 3).

## 4. Discussion

This study is the first attempt to unveil the expression characteristics of genomic-wide microRNAs in the colon tissues of goats. Although the microRNA profiles in the intestine tissues of bovine [18] and sheep [4] have already been described, our study also delineated the temporal variation of colonic microRNAs.

By analyzing the high-throughput sequence data, 39 DEmiRNAs, including 13 novel microRNAs, were observed among the three developmental stages, which participated in the bacterial invasion of the epithelial cell pathway, ErbB signaling pathway, sphingolipid signaling pathway, etc. The enrichment of these pathways reflected that DEmiRNAs played a major role in the regulation of the innate immune response to pathogenic infection, cell proliferation, cell differentiation, cell motility, and cell survival of the colonic epithelium. Among these microRNAs, chi-miR-145, which belongs to the bacterial invasion of epithelial cells and the Axon guidance pathways, was reported to promote M2 macrophage polarization by targeting IL-16 expression [39]. It has been shown that human miR-150 was associated with inflammatory bowel disorders and pain and interacted with a protein kinase AKT2 through which it might affect inflammatory pathways [40]. MIR-150 deficiency reduced the intraepithelial lymphocyte population in the small intestine and increased susceptibility to anticancer drug-induced mucositis [41]; hence, miR-150-mediated intraepithelial lymphocyte generation was crucial for maintaining intestinal integrity.

Eighty-six highly expressed microRNAs were also identified in the colon of goats. Among these highly expressed microRNAs, miR-148a, miR-26a, miR-Let-7f, miR-Let-7g, miR-Let-7i, miR-10a, miR-27b, miR-127, miR-99a, and miR-145 were also defined as high-expression microRNAs in the rumen during pregnancy and after birth in goats [17]. In particular, as one of the largest microRNAs among vertebrates, the Let-7 family of microRNAs are critical for promoting cell differentiation, regulating metabolism, and inhibiting cellular proliferation. Nine members of Let-7 were identified in the present study, including chi-let-7a, chi-let-7b, chi-let-7c, chi-let-7d (-3p and -5p), chi-let-7e, chi-let-7f, chi-let-7g, and chi-let-7i (Table 1). Intestinal Let-7 in *Caenorhabditis elegans* has been shown to be involved in the regulation of the innate immune response to a *Pseudomonas aeruginosa PA14* infection by suppressing both the expression and function of the OB domain-containing protein SDZ-24 [42]. Apart from the common shared 81 KEGG biological pathways with DEmiRNAs, these microRNAs are also involved in the other 40 KEGG pathways, including the tight junction, phosphatidylinositol signaling system, ubiquitin mediated proteolysis, and nucleotide excision repair, indicating that DEmiRNAs have wider physiological functions on the colon epithelium barrier, phospholipid metabolism, protein ubiquitination, and DNA damage repair. This kind of microRNA should not be neglected in the future. Whether HEmiRNAs interact with DEmiRNAs needs to be investigated.

During the eight profiles of dynamic microRNAs, approximately half of these microRNA were affected by age. Specifically, chi-miR-493 expression continued to decrease from D10 to D31. Previous researchers reported that human miR-493 was an important regulator for colon [43] and bladder [44] cancers as it targeted genes’ insulin-like growth factor receptor (*IGF1R*), MAP kinase kinase 7 (*MKK7*), ras homolog family member C (*RhoC*), and frizzled class receptor 4 (*FZD4*) with central roles in these cancers. Bovine miR-493 was the most significant differentially expressed microRNA between the preweaning (D33) and postweaning (D96) period [16]. However, the function of miR-493 in the ruminant animals is still unclear. Additionally, chi-miR-130b expression also deceased from D10 to D31. MIR-130b’s role in the definitive host response in canine echinococcosis was reflected by its upregulation expression in the distal segments of the small intestine of canines [45]. Furthermore, chi-miR-29a expression increased from D10 to D31. The expression of miR-29 was correlated with the number of Bifidobacterium and Lactobacillus spp. in different ages of calves, revealing a potentially fundamental regulatory mechanism by which probiotics have an effect [15]. Pig miR-29a upregulation was associated with intrauterine growth restriction and impaired intestinal barrier function by downregulating the extracellular matrix and tight junction protein expression [46]. Therefore, dynamic microRNAs during colon development also need more attention.

## 5. Conclusions

The genome-wide expression profile of microRNAs and temporal variations of microRNAs regulating the development of the colon were identified in the young goats. Biological pathways of DEmiRNAs and HEmiRNAs were overlapping, but the latter one owns broader physiological functions on the colon epithelium barrier, phospholipid metabolism, protein ubiquitination, and DNA damage repair. Nearly half of the dynamic microRNAs were altered during the development of the colon, and this type of molecule deserves more attention.

## Figures and Tables

**Table 1:** Key miRNAs in the colon of goats at the preregurgitation stage.

miRNA	Sequences	D10_1 (norm)	D10_2 (norm)	D10_3 (norm)	D10_4 (norm)	D10_5 (norm)	D25_1 (norm)	D25_2 (norm)	D25_3 (norm)	D25_4 (norm)	D25_5 (norm)	D31_1 (norm)	D31_2 (norm)	D31_3 (norm)	D31_4 (norm)	D31_5 (norm)
chi-miR-145-5p	GTCCAGTTTTCCCAGGAATCCCT	413,928.08	273,737.54	305,373.85	259,564.08	158,024.67	793,964.42	638,968.89	541,464.83	631,723.29	709,422.09	752,564.55	708,834.75	619,420.59	683,159.93	806,093.4
chi-miR-150	TCTCCCAACCCTTGTACCAGTG	7238.49	7862.5	10,018.71	5326.91	3185.05	7577.22	4807.91	8794.09	8113.97	11,637.64	8922.71	9453.68	18,551.5	17,071.68	17,578.87
chi-miR-493-5p	TTGTACATGGTAGGCTTTCATT	4999.65	7253.76	6637.88	4949.41	4818.39	2541.94	3025.21	3008.33	3475.73	1959.88	2622.32	3930.38	3009.98	2527.48	2423.76
chi-let-7a-5p	TGAGGTAGTAGGTTGTGTGGTT	273,674.8	308,517.33	296,359.97	317,003.02	330,373.78	311,181.55	521,287.55	306,841.98	352,508.62	303,980.85	399,522.55	392,244.07	339,988.32	255,973.03	245,615.7
chi-let-7b-5p	TGAGGTAGTAGGTTGTGTGGTT	176,240.7	173,168.72	187,498.46	222,957.97	151,793.56	227,617.78	245,458.22	194,446.03	201,309.73	256,727.74	242,442.15	267,225.59	239,427.19	275,758.49	21,3294.5
chi-let-7c-5p	TGAGGTAGTAGGTTGTATGGTT	19,998.78	19,328.18	22,494.28	25,363.75	15,374.01	17,784.34	27,865.21	14,034.72	20,558.67	15,736.86	22,688.46	29,583.58	24,529.86	19,915.93	15,916.33
chi-let-7d-5p	AGAGGTAGTAGGTTGCATAGTT	17,727.73	20,104.59	18,721.97	21,742.38	19,750.87	19,055.95	18,598.87	19,642.49	19,593.06	21,274.85	22,562.2	20,098.75	19,962.66	19,492.2	16,406.29
chi-let-7d-3p	CTATACGACCTGCTGCCTTTCT	4844.61	7089.73	6341.19	6762.08	5883.03	3801.47	14,170.87	6507.76	5145.36	9214.98	5528.73	7017.13	6903.8	7994.49	5557.47
chi-let-7e-5p	TGAGGTAGGAGGTTGTATAGTT	32,213.65	40,306.38	40,948.79	39,775.16	33,621.36	32,349.5	34,276.18	31,642.95	39,923.47	33,233.69	41,543.94	50,541.23	36,741.77	29,093.51	27,127.68
chi-let-7f-5p	TGAGGTAGTAGATTGTATAGTT	31,245.66	31,577.41	24,545.72	22,108.92	40,557.5	31,878.85	68,705.39	37,339.47	31,208.06	27,579.35	28,455.72	29,908.99	24,931.13	17,577.23	23,287.28
chi-let-7g-5p	TGAGGTAGTAGTTTGTACAGTT	20,827.40	27,945.82	19,867.21	20,364.65	31,014.77	20,261.49	53,386.92	22,753.72	24,312.68	20,075.23	24,907.09	27,355.42	20,522.19	15,088.47	16,710.72
chi-let-7i-5p	TGAGGTAGTAGTTTGTGCTGTT	32,712.21	31,512.86	26,314.27	25,278.73	34,437.59	31,221.13	79,115.93	37,108.08	29,293.7	28,501.77	28,455.99	31,761.57	27,101.97	22,835.44	20,823.23
chi-miR-29a-5p	ACTGATTTCTTTTGGTGTTCAGAGT	6.20	6.38	4.92	0.00	11.53	30.49	0.00	26.32	14.12	0.00	47.35	12.27	9.38	28.05	28.19

Norm: the normalized value.

## Data Availability

All the sequencing data was deposited in the publicly available NCBI’s Sequence Read Archive (https://www.ncbi.nlm.nih.gov/sra accessed on 15 May 2022). The data is accessible through accession number PRJNA744769 (http://www.ncbi.nlm.nih.gov/bioproject/744769 accessed on 15 May 2022).

## Supplementary Materials

The following supporting information can be downloaded at: https://www.mdpi.com/article/10.3390/ani12121533/s1: Table S1: primer sequences for real-time PCR; Table S2: overview of the reads from raw data to cleaned sequences of small RNA sequences; Table S3: information of the highly expressed miRNAs in the goat colon of three different periods; Table S4: the dynamic miRNAs in goat colon from D10 to D31; Table S5: the identified DEmiRNAs in the goat colon; Table S6: the enriched KEGG pathway of differentially expressed miRNAs in goat colon from D10 to D31; Table S7: the enriched KEGG pathway of highly expressed miRNAs in goat colon from D10 to D31.
