# Supplementary material for: Developmental Alterations of Colonic microRNA Profiles Imply Potential Biological Functions in Kid Goats
Source: Animals (Basel). 2022 Jun 14;12(12):1533. doi: 10.3390/ani12121533 (PMC9219484; doi:10.3390/ani12121533)
Supplement: Supplementary file 1 [file animals-12-01533-s001.zip › animals-1723041-supplementary - FC.pdf]

Table S1 Primer sequences for real-time PCR

miRNA	Primer	Sequence
U6	Up primer	CGCTTCGGCAGCACATATAC
	Down primer	TCACGAATTTGCGTGTCAT
chi-let-7b-5p	Up primer	CCGTGAGGTAGTAGGTTGTGTGGTT
chi-miR-107-3p	Up primer	CGAGCAGCATTGTACAGGGCTAT
chi-miR-30b-5p	Up primer	CCGCTGTAAACATCCTACACTCAGCT
chi-miR-1271-3p	Up primer	CGTCACAGTGAACCGGTCTCTTT
chi-miR-92a-5p	Up primer	AGGTTGGGATCAGTTGCAATGCT
chi-miR-412-3p	Up primer	TATACGCTTCACCTGGTCCACTAGC

Table S2: Overview of reads from raw data to cleaned sequences of small RNA sequences.

Type	D10_1		D10_2		D10_3		D10_4		D10_5	
	Uniq	% of uniq	Uniq	% of uniq	Uniq	% of uniq	Uniq	% of uniq	Uniq	% of uniq
Raw reads	863442	100.00	1023031	100.00	1349392	100.00	1307004	100.00	949935	100.00
3ADT&length filter	476498	55.19	547130	53.48	793297	58.79	848030	64.88	544110	57.28
Junk reads	6163	0.71	7110	0.69	6798	0.50	6861	0.52	5921	0.62
Rfam	11680	1.35	12669	1.24	14641	1.09	14884	1.14	12709	1.34
mRNA	18716	2.17	17626	1.72	21711	1.61	18720	1.43	14504	1.53
Repeats	1324	0.15	1582	0.15	1753	0.13	1931	0.15	1634	0.17
Valid reads	353058	40.89	440934	43.10	515858	38.23	421672	32.26	375327	39.51

Type	D25_1		D25_2		D25_3		D25_4		D25_5	
	Uniq	% of uniq	Uniq	% of uniq	Uniq	% of uniq	Uniq	% of uniq	Uniq	% of uniq
Raw reads	998568	100.00	917367	100.00	1489136	100.00	1064340	100.00	1187114	100.00
3ADT&length filter	518623	51.94	512348	55.85	759610	51.01	626571	58.87	539346	45.43
Junk reads	8583	0.86	4263	0.46	12349	0.83	6927	0.65	9931	0.84
Rfam	11863	1.19	10411	1.13	16740	1.12	14807	1.39	14443	1.22
mRNA	16088	1.61	15852	1.73	27692	1.86	20096	1.89	22255	1.87
Repeats	1305	0.13	1338	0.15	1686	0.11	1561	0.15	1697	0.14
Valid reads	445955	44.66	376668	41.06	676188	45.41	398934	37.48	604124	50.89

Type	D31_1		D31_2		D31_3		D31_4		D31_5	
	Uniq	% of uniq	Uniq	% of uniq	Uniq	% of uniq	Uniq	% of uniq	Uniq	% of uniq
Raw reads	1215693	100.00	1090285	100.00	1058913	100.00	1546792	100.00	1800286	100.00
3ADT&length filter	601086	49.44	550849	50.52	502707	47.47	685778	44.34	1028346	57.12
Junk reads	9399	0.77	6572	0.60	7709	0.73	14981	0.97	13989	0.78
Rfam	15045	1.24	14985	1.37	11486	1.08	18371	1.19	16436	0.91
mRNA	22095	1.82	19174	1.76	18500	1.75	35054	2.27	30393	1.69
Repeats	1606	0.13	1488	0.14	1442	0.14	2103	0.14	1900	0.11
Valid reads	570984	46.97	501582	46.00	521044	49.21	796524	51.50	714684	39.70

Table S3: Information of the highly expressed miRNAs in the goat colon of three different periods.

miR_name	Sequence	D10_1 (norm)	D10_2 (norm)	D10_3 (norm)	D10_4 (norm)	D10_5 (norm)	D25_1 (norm)	D25_2 (norm)	D25_3 (norm)	D25_4 (norm)	D25_5 (norm)	D31_1 (norm)	D31_2 (norm)	D31_3 (norm)	D31_4 (norm)	D31_5 (norm)
chi-let-7f-5p	TGAGGTAGTAGATTGTATAGTT	31245.66	31577.41	24545.72	22108.92	40557.50	31878.85	68705.39	37339.47	31208.06	27579.35	28455.72	29908.99	24931.13	17577.23	23287.28
chi-let-7d-5p	AGAGGTAGTAGGTTGCATAGTT	17727.73	20104.59	18721.97	21742.38	19750.87	19055.95	18598.87	19642.49	19593.06	21274.85	22562.20	20098.75	19962.66	19492.20	16406.29
chi-let-7d-3p	CTATACGACCTGCTGCCTTTCT	4844.61	7089.73	6341.19	6762.08	5883.03	3801.47	14170.87	6507.76	5145.36	9214.98	5528.73	7017.13	6903.80	7994.49	5557.47
chi-let-7e-5p	TGAGGTAGGAGGTTGTATAGTT	32213.65	40306.38	40948.79	39775.16	33621.36	32349.50	34276.18	31642.95	39923.47	33233.69	41543.94	50541.23	36741.77	29093.51	27127.68
chi-let-7a-5p	TGAGGTAGTAGGTTGTGTGGTT	273674.80	308517.33	296359.97	317003.02	330373.78	311181.55	521287.55	306841.98	352508.62	303980.85	399522.55	392244.07	339988.32	255973.03	245615.70
chi-let-7i-5p	TGAGGTAGTAGTTTGTGCTGTT	32712.21	31512.86	26314.27	25278.73	34437.59	31221.13	79115.93	37108.08	29293.70	28501.77	28455.99	31761.57	27101.97	22835.44	20823.23
chi-let-7g-5p	TGAGGTAGTAGTTTGTACAGTT	20827.40	27945.82	19867.21	20364.65	31014.77	20261.49	53386.92	22753.72	24312.68	20075.23	24907.09	27355.42	20522.19	15088.47	16710.72
chi-let-7c-5p	TGAGGTAGTAGGTTGTATGGTT	19998.78	19328.18	22494.28	25363.75	15374.01	17784.34	27865.21	14034.72	20558.67	15736.86	22688.46	29583.58	24529.86	19915.93	15916.33
chi-let-7b-5p	TGAGGTAGTAGGTTGTGTGGTT	176240.70	173168.72	187498.46	222957.97	151793.56	227617.78	245458.22	194446.03	201309.73	256727.74	242442.15	267225.59	239427.19	275758.49	213294.50
chi-miR-10a-5p	TACCCTGTAGATCCGAATTTGT	61275.44	62140.88	37246.00	27586.79	28542.14	68528.90	324180.62	59130.15	66710.19	48078.30	40605.32	71483.46	62414.76	28559.75	54096.73
chi-miR-10b-5p	TACCCTGTAGAACCGAATTTGT	83940.80	144259.68	65070.37	50191.29	107257.26	112533.55	496302.40	115090.28	122741.97	71568.14	91293.33	124972.55	94762.61	36804.97	109924.35
chi-miR-15b-5p	TAGCAGCACATCATGGTTTACA	11208.64	13415.82	14560.07	14409.52	9032.59	15375.47	6677.44	11321.83	15858.47	14897.83	17803.06	12090.30	17598.22	18493.66	16968.02
chi-miR-16a-5p	TAGCAGCACGTAAATATTGGAG	35752.62	34798.93	33487.76	35944.45	38726.32	45266.57	15268.46	41279.50	42988.96	37575.85	54593.67	42767.59	45125.23	32024.41	37342.72
chi-miR-16b-5p	TAGCAGCACGTAAATATTGGGGT	35517.99	35954.43	34295.88	29418.16	28622.87	45564.78	17434.25	44566.09	45192.76	43874.74	51678.97	43648.03	46939.85	39158.87	42668.67
chi-miR-21-5p	TAGCTTATCAGACTGATGTTGAC	74169.93	45746.10	38806.10	31978.46	75424.49	101040.06	93761.21	161189.87	46105.82	59979.95	50154.74	40877.89	51023.21	41172.61	63962.63

chi-miR-22-3p	AAGCTGCCAGTTGAAGAACTGT	22087.56	16599.82	18985.88	22805.15	23012.22	26298.75	19557.26	29765.13	22556.97	19791.59	19415.33	15278.96	17003.36	14130.40	17773.66
chi-miR-23a	ATCACATTGCCAGGGATTTCCA	82978.49	65670.25	78868.63	66701.42	64283.02	110036.40	51555.06	95555.34	103646.03	80819.71	113583.50	95078.51	95547.00	103799.89	87428.33
chi-miR-23b-3p	ATCACATTGCCAGGGATTACCAC	133104.20	131714.13	156157.08	138250.22	138852.26	147873.90	93665.43	115966.83	192298.40	144988.38	209574.50	204187.50	165829.17	163783.52	135452.42
chi-miR-24-3p	TGGCTCAGTTCAGCAGGAACAGT	40784.83	32631.91	36585.00	38759.81	38466.37	45744.37	22724.22	33964.00	41356.99	37496.74	43184.03	41366.71	39540.64	36327.18	36777.58
chi-miR-25-3p	CATTGCACTTGTCTCGGTCTGA	50610.48	55247.98	68050.82	63119.16	74336.78	45852.03	58881.21	58094.53	60706.49	53001.72	72787.13	76805.29	63806.22	47145.46	46415.37
chi-miR-26a-5p	TTCAAGTAATCCAGGATAGGCT	290462.96	208818.18	253956.68	249888.01	277753.75	279325.25	389337.65	262252.32	309618.50	212521.57	329445.51	350132.17	322036.37	179724.71	214181.88
chi-miR-26b-5p	TTCAAGTAATTCAGGATAGGTT	18240.92	15065.23	13079.89	12754.99	17737.36	17301.93	9211.64	13334.40	17061.57	12223.52	16673.81	13915.83	13812.30	11225.95	13402.87
chi-miR-27b-3p	TTCACAGTGGCTAAGTTCTGC	196235.00	194481.06	185781.28	197366.55	304629.28	159991.92	508343.24	140905.10	179853.30	151888.49	186223.19	197298.71	174079.39	126295.61	124029.06
chi-miR-27a-3p	TTCACAGTGGCTAAGTTCTGCT	20014.11	13022.15	15879.62	16467.05	19137.81	24138.39	12577.18	18811.44	15647.86	16372.19	18301.44	14880.73	17844.05	17755.05	16910.35
chi-miR-28-3p	CACTAGATTGAGAGCTCCTGGA	15879.60	16288.16	15023.14	14849.37	15532.23	14658.05	47860.70	18328.23	14833.64	18357.78	17489.08	17543.77	18095.50	18356.02	15894.97
chi-miR-29a-3p	TAGCACCATCTGAAATCGGTTA	10120.24	8025.62	7902.52	7020.55	9810.67	10653.65	7762.89	14072.20	10641.34	13441.28	14468.05	11016.40	14605.13	10578.95	12817.64
chi-miR-30b-5p	TGTAAACATCCTACACTCAGCT	19374.29	14295.20	14427.30	13312.17	14965.75	19186.47	7321.04	11105.24	17476.32	12181.99	19552.38	18930.96	19215.80	9939.85	16795.87
chi-miR-30c-5p	TGTAAACATCCTACACTCTCAGCT	112280.76	110130.08	88273.05	74750.73	84263.23	115693.35	98426.09	99048.38	147437.44	111663.05	132038.19	139418.72	128165.07	88494.73	136830.90
chi-miR-30f-5p	TGTAAACACCCTACACTCTCAGCT	17991.30	19253.00	14830.95	17673.80	12309.91	26754.16	27423.58	18099.59	21949.83	23521.49	26039.67	27240.92	26595.31	29144.06	39820.30
chi-miR-30a-5p	TGTAAACATCCTCGACTGGAAGCT	23002.32	17309.70	14127.74	13110.95	16340.47	32243.43	91366.83	23928.98	20376.10	19140.44	21426.72	23769.63	20341.73	14469.68	18929.16
chi-miR-30e-5p	TGTAAACATCCTTGACTGGAAGCT	14812.38	9288.64	7471.00	5836.47	11345.52	19004.97	40769.52	18016.49	13041.65	12156.78	12047.57	10962.61	9515.95	7526.78	10695.47
chi-miR-92b	TATTGCACTTGTCCCGGCCTGT	66963.48	64004.44	73273.27	81684.55	83213.67	60723.50	92643.19	78797.46	65935.38	84666.29	80895.82	67022.26	88932.19	115874.02	86577.84

chi-miR-92b	TATTGCACTTGTCCCGGCCTGT	66963.48	64004.44	73273.27	81684.55	83213.67	60723.50	92643.19	78797.46	65935.38	84666.29	80895.82	67022.26	88932.19	115874.02	86577.84
chi-miR-93-5p	CAAAGTGCTGTTCGTGCAGGTAG	25222.55	24945.29	28047.30	20722.68	26230.98	27005.69	17167.99	31709.26	34650.26	27812.06	38161.16	33031.27	25425.28	22381.76	22610.08
chi-miR-99a-5p	AACCCGTAGATCCGATCTTGT	167177.69	157458.61	143857.86	162109.08	121271.04	147777.19	539839.18	131113.75	158757.69	127848.85	152753.89	228667.24	178494.90	118130.17	128140.14
chi-miR-99b-5p	CACCCGTAGAACCGACCTTGCG	115796.13	151188.12	119575.36	107430.33	84552.90	85275.86	563668.01	93788.32	91129.15	129596.13	89483.79	139628.22	127225.87	142445.46	141938.13
chi-miR-100-5p	AACCCGTAGATCCGATCTTGT	167177.69	157458.61	143857.86	162109.08	121271.04	147777.19	539839.18	131113.75	158757.69	127848.85	152753.89	228667.24	178494.90	118130.17	128140.14
chi-miR-103-3p	AGCAGCATTGTACAGGGCTATGA	54320.17	57872.46	56674.46	47463.22	68170.29	61571.45	80161.79	76525.80	67447.34	57381.78	68465.74	62166.61	55252.00	42961.93	39428.59
chi-miR-106b-3p	CCGCACTGTGGGTACTTGCTGC	4595.50	6311.50	5110.16	5189.74	5193.67	3475.63	10996.25	4867.47	4364.08	5277.43	4087.57	4257.84	5286.22	5549.41	5595.06
chi-miR-125b-5p	TCCCTGAGACCCTAACTTGTGA	325308.05	366963.89	357392.61	410646.95	214696.39	306550.07	811122.60	315553.84	354191.78	503747.73	372845.10	489928.83	482108.30	482556.10	573169.90
chi-miR-125a-5p	TCCCTGAGACCCTTTAACCTGT	76423.23	73473.52	69836.71	83714.88	44604.91	77510.95	172388.68	69580.32	81558.53	127075.58	86828.24	102271.16	113921.17	135995.69	121615.98
chi-miR-126-3p	TCGTACCGTGAGTAATAATGCG	22155.78	20079.99	16552.50	20647.86	22652.90	27067.62	18944.30	24143.32	23704.17	17441.13	23834.32	22610.33	18744.79	14363.60	20422.97
chi-miR-127-3p	TCGGATCCGTCTGAGCTTGGCT	159180.53	198602.76	190836.53	179957.46	160685.38	119067.03	490227.71	130713.27	127489.04	127811.27	107991.23	172715.17	160027.84	175085.30	122465.19
chi-miR-133a-3p	TTGGTCCCCTTCAACCAGCTGT	63330.29	46415.43	57773.53	52680.17	23719.76	74072.95	35867.11	47496.95	69303.46	66436.07	58866.47	83550.64	51673.43	76644.15	66789.64
chi-miR-140-3p	ACCACAGGGTAGAACCACGGAC	31624.83	30640.77	28587.41	29835.90	28506.65	28652.04	119940.99	35911.89	26175.07	39225.23	32540.24	44156.20	41441.58	35936.18	30350.38
chi-miR-143-5p	GGTGCAGTGCTGCATCTCTGG	7447.28	7232.80	7442.73	7400.31	5505.08	9562.75	14938.35	8685.79	6923.23	9599.64	7505.64	8307.13	5753.48	5523.99	6399.85
chi-miR-143-3p	TGAGATGAAGCACTGTAGCTC	1265442.91	978624.76	1185189.08	1223970.31	1004382.33	1748637.71	3312785.67	1488402.37	1183807.11	1378149.54	1434338.82	1821693.24	1234695.89	681185.63	823166.51
chi-miR-145-5p	GTCCAGTTTTCCCAGGAATCCCT	413928.08	273737.54	305373.85	259564.08	158024.67	793964.42	638968.89	541464.83	631723.29	709422.09	752564.55	708834.75	619420.59	683159.93	806093.40
chi-miR-148b-3p	TCAGTGCATCACAGAACTTTGT	11716.15	16633.54	15764.87	14707.66	18941.74	12204.72	29471.89	13071.93	15800.23	11653.46	18318.89	13771.45	16121.38	10127.90	9856.51

chi-miR-148a-3p	TCAGTGCACTACAGAACTTTGT	17993.37	13478.70	12347.17	9831.94	19812.08	20308.81	63178.32	25952.08	16426.19	14153.24	14891.68	16023.04	14462.52	7909.01	11173.04
chi-miR-150	TCTCCCAACCCTTGTACCAGTG	7238.49	7862.50	10018.71	5326.91	3185.05	7577.22	4807.91	8794.09	8113.97	11637.64	8922.71	9453.68	18551.50	17071.68	17578.87
chi-miR-151-5p	TCGAGGAGCTCACAGTCTAGT	39841.64	59860.41	50657.82	56378.05	60594.93	37213.45	112601.43	42147.40	45274.54	42107.69	51792.35	57195.83	47958.81	36867.21	40825.43
chi-miR-151-3p	CTAGACTGAAGCTCCTTGAGG	12902.76	17417.23	12956.13	14128.38	16357.32	10902.31	32350.25	13511.90	12716.90	13176.27	12666.40	16005.58	13610.57	9682.10	13138.88
chi-miR-181b-5p	AACATTCATTGCTGTCGGTGGGTT	19062.65	19039.31	21285.24	15153.75	14940.91	23146.58	25074.54	22894.49	24424.85	21651.10	26571.69	32499.99	22415.31	21861.88	17587.42
chi-miR-186-5p	CAAAGAATTCTCCTTTTGGGCTT	23493.29	20199.36	17199.16	16012.47	21252.01	30013.52	38970.22	26861.34	29226.04	23320.76	26362.78	22464.06	24708.44	16435.20	21047.49
chi-miR-191-5p	CAACGGAATCCCAAAAGCAGCTG	91284.71	82650.08	87009.65	94805.14	102237.48	99899.62	322962.36	134048.00	98190.04	136355.82	129039.59	101344.48	138137.96	109419.87	103426.25
chi-miR-192-5p	CTGACCTATGAATTGACAGCC	88187.97	103572.54	73356.46	81439.12	199865.94	56505.68	219895.65	64759.11	82610.43	56386.51	65916.93	58206.50	58216.93	30809.32	48731.06
chi-miR-193b-3p	AACTGGCCCACAAAGTCCCGCT	17306.01	10998.20	12463.55	16264.13	7850.84	14316.02	14785.11	16803.76	13027.53	14312.44	13013.60	10673.85	16444.15	27350.80	18504.98
chi-miR-194	TGTAACAGCAACTCCATGTGGA	130256.56	105981.50	95792.41	116805.42	194800.92	89265.02	67951.75	86648.06	115745.20	86480.81	104781.64	78949.30	89398.51	55807.98	91344.19
chi-miR-196b	TAGGTAGTTTCCTGTTGTTGGG	10525.42	12794.33	8681.95	12941.47	18458.21	7630.57	5245.92	5897.07	12588.65	9848.83	16154.25	9389.51	10613.73	5947.43	8258.89
chi-miR-197-3p	TTCACCACCTTCTCCACCCAGC	13850.59	15759.62	17677.80	17327.47	15088.62	11520.65	21781.80	13946.60	14717.15	18705.85	14222.18	15416.73	15342.62	28061.80	21572.91
chi-miR-199c-5p	CCCAGTGTTCAGACTACCTGTTC	45779.83	38312.81	29670.09	33933.39	40887.54	41187.37	174491.90	42558.79	36835.24	31436.15	34419.56	37742.07	44641.08	22696.49	27605.82
chi-miR-199a-3p	ACAGTAGTCTGCACATTGGTT	30784.49	33963.74	25116.43	28068.01	33597.85	26143.45	28798.91	23614.23	24792.54	19997.77	23068.89	27623.10	26479.90	17244.38	23479.37
chi-miR-199a-5p	CCCAGTGTTCAGACTACCTGTTC	45779.83	38312.81	29670.09	33933.39	40887.54	41187.37	174491.90	42558.79	36835.24	31436.15	34419.56	37742.07	44641.08	22696.49	27605.82
chi-miR-199a-3p	ACAGTAGTCTGCACATTGGTT	30784.49	33963.74	25116.43	28068.01	33597.85	26143.45	28798.91	23614.23	24792.54	19997.77	23068.89	27623.10	26479.90	17244.38	23479.37
chi-miR-200b	TAATACTGCCTGGTAATGATGAC	250913.83	231979.19	188729.36	185024.20	398711.61	180883.34	373249.36	242647.06	311846.43	225913.40	305546.82	216668.48	270118.99	169230.80	192533.36

chi-miR-200a	TAACACTGTCTGGTAACGATGTT	50695.24	37807.96	33054.19	28554.91	77658.46	40612.38	28076.13	43769.64	50536.97	34507.48	49615.25	34638.34	32443.55	23711.69	30001.39
chi-miR-200c	TAATACTGCCGGGTAATGATGGA	142744.83	132646.37	106194.69	100223.31	177858.04	111039.17	113744.99	126130.08	133042.68	145257.84	127252.06	102610.88	139025.57	129327.63	134852.68
chi-miR-214-3p	ACAGCAGGCACAGACAGGCAGT	29364.29	34544.68	38456.13	43015.44	23157.72	24843.90	13465.97	17963.47	25620.88	30410.73	30744.40	32777.43	36853.43	50807.38	41848.93
chi-miR-215-5p	ATGACCTATGAATTGACAGACA	2464.17	1118.59	1260.12	1635.26	4034.99	1610.15	19151.18	3194.47	2159.10	1939.11	2397.22	2237.44	1554.72	1038.87	1200.77
chi-miR-221-3p	AGCTACATTGTCTGCTGGGTTTC	16843.98	14266.04	12956.13	11271.64	15225.25	15008.67	11370.41	18014.61	16745.64	12938.95	20840.70	14848.64	16023.80	15299.02	12810.81
chi-miR-222-3p	AGCTACATCTGGCTACTGGGTCTCT	15056.84	10456.90	9171.25	7226.87	9756.55	14923.87	22731.89	20286.65	13787.62	16338.57	17420.14	11729.82	15184.99	19621.08	17206.38
chi-miR-224-5p	CAAGTCACTAGTGGTTCCGTTT	9299.54	10005.82	9085.19	8088.42	10942.74	7452.41	21543.00	9239.32	9649.45	7582.41	9872.13	11455.21	8354.37	7009.97	6885.12
chi-miR-320-3p	AAAAGCTGGGTTGAGAGGGCGA	49012.49	53214.01	56629.79	59097.06	56655.76	51449.44	58260.59	64067.57	56247.10	70089.83	83169.28	84691.54	69709.82	109690.77	65181.78
chi-miR-342-3p	TCTCACACAGAAATCGCACCCATC	57838.63	49887.85	45589.87	44033.43	40630.25	73595.15	41295.64	56338.42	73986.99	61717.34	70126.60	60650.60	79353.38	63958.52	75358.66
chi-miR-361-5p	TTATCAGAATCTCCAGGGGTAC	12656.75	9718.77	11030.08	11624.20	10516.88	12854.50	16515.45	12337.14	13042.82	11626.76	13201.33	9756.60	14807.80	11435.47	11194.40
chi-miR-365-3p	TAATGCCCCTAAAAATCCTTAT	12878.98	8581.49	8618.02	10524.58	5145.76	15033.44	6987.75	12211.55	13105.18	11095.75	12110.70	8383.56	15200.00	16126.61	15834.31
chi-miR-378-3p	ACTGGACTTGGAGTCAGAAGGC	117211.16	165700.20	128123.72	129840.99	161087.28	116524.14	399160.97	134281.89	132729.70	142785.25	177164.26	151023.97	126942.51	134739.40	150798.48
chi-miR-380-3p	TATGTAATGTGGTCCACGTCT	11687.21	9819.92	12592.23	14123.85	9256.17	8922.50	6402.88	6379.91	8004.54	7093.92	7232.36	7372.89	11338.07	8198.75	7904.34
chi-miR-409-5p	AGGTTACCCGAGCAACTTTGCAT	4650.28	4406.02	3750.46	2998.44	3530.17	3809.09	18641.65	5020.90	3599.28	3712.10	2649.73	3177.34	3644.25	3494.46	2604.88
chi-miR-409-3p_R+1	GAATGTTGCTCGGTGAACCCCT	16353.00	17111.05	18988.33	16487.46	14681.40	12701.11	32168.92	20048.99	13080.48	13335.47	10898.80	12287.52	12482.76	15791.72	12090.60
chi-miR-423-5p	TGAGGGGCAGAGAGCGAGACTTT	25229.79	26387.84	27903.87	23292.61	25257.72	27293.42	59727.87	42902.49	30520.32	45913.73	39892.21	36880.50	40463.90	43376.60	33302.98

chi-miR-423-3p	AGCTCGGTCTGAGGCCCCTCAGT	45392.74	61522.12	47283.15	41076.94	44643.06	42981.40	93362.78	68795.15	52951.39	80568.55	60626.60	59735.24	60208.88	84922.68	73601.28
chi-miR-455-3p_R-1	GCAGTCCATGGGCATATACAC	10201.89	14532.13	14596.95	17046.33	10243.62	14674.25	9845.67	13137.36	12152.12	15240.96	17659.36	14293.77	12987.55	19230.95	16381.95
chi-miR-490	CAACCTGGAGGACTCCATGCTGT	7649.87	4271.15	4968.37	2670.82	1683.91	4637.99	17129.68	5105.13	4242.89	5685.82	3089.14	5528.02	3115.06	4993.60	3983.78
chi-miR-500-3p	ATGCACCTGGGCAAGGATTCTGA	2681.23	3180.81	3125.93	2657.22	3243.16	2369.49	14198.33	3253.88	3192.17	3338.81	3150.61	3314.64	2905.83	2749.28	2346.01
chi-miR-532-5p	CATGCCTTGAGTGTAGGACCGT	5591.92	5445.79	6647.72	7069.29	8028.28	5440.20	12748.30	7238.78	5364.21	5678.90	6710.72	6678.35	4513.09	4388.68	4420.35
chi-miR-3431-5p	CCTCAGTCAGCCTTGTGGATGT	12515.15	12208.38	12572.56	10588.07	17275.57	9865.72	15921.64	12849.31	11335.55	11145.19	13085.04	14699.54	11338.07	11818.58	9932.54

Table S4: The dynamic miRNAs in goat colon from D10 to D31

	miRNA	D10	D25	D31	*P*-values
Gene Table for Profile 0 (0.0, -1.0, -2.0)					0.024
1	CHI-MIR-1197-3P	0	-19.41	-36.53	
2	CHI-MIR-758_R-1	0	-273.70	-465.43	
3	CHI-MIR-411A-3P	0	-543.55	-912.93	
4	CHI-MIR-493-3P	0	-179.44	-255.48	
5	CHI-MIR-382-3P_R+1	0	-39.17	-139.70	
6	CHI-MIR-412-5P	0	-109.14	-269.07	
7	CHI-MIR-130B-5P	0	-628.74	-1169.07	
8	CHI-MIR-665_R+2	0	-202.00	-388.48	
9	CHI-MIR-3959-3P	0	-28.94	-62.77	
10	CHI-MIR-379-5P	0	-397.83	-1442.10	
11	CHI-MIR-381_1SS4AG	0	-59.05	-204.99	
12	CHI-MIR-301A-5P_R+1	0	-8.89	-14.47	
Gene Table for Profile 1 (0.0, -1.0, -1.0)					0.023
1	CHI-MIR-493-5P	0	-2929.6	-2,829.03	
2	CHI-MIR-124A_R-1	0	-493.19	-289.55	
3	CHI-MIR-410-3P	0	-865.49	-1,140.76	
4	CHI-MIR-323B	0	-593.06	-792.81	
5	CHI-MIR-411B-3P_1SS6CA	0	-29.82	-21.65	
6	CHI-MIR-1271-3P	0	-18.92	-14.78	
7	CHI-MIR-134_R-1	0	-45.65	-60.83	
8	CHI-MIR-592	0	-18.00	-15.46	
9	CHI-MIR-380-3P	0	-4,135.12	-3,086.59	
10	CHI-MIR-382-5P	0	-705.72	-663.6	
11	CHI-MIR-1306-3P_1SS22GT	0	-100.07	-71.03	
12	CHI-MIR-379-3P	0	-613	-638.01	
13	CHI-MIR-412-3P_L-1	0	-20.49	-21.53	
14	CHI-MIR-128-3P	0	-2,059.22	-2,097.99	
15	CHI-MIR-496-5P	0	-7.67	-5.96	
16	CHI-MIR-1307-5P_R+5	0	-201.20	-115.13	
17	CHI-MIR-496-3P_R+1	0	-35.19	-46.13	
18	CHI-MIR-107-3P	0	-1,473.39	-1,902.38	

19	CHI-MIR-199A-3P	0	-5,636.73	-6,726.98	
20	CHI-MIR-432-5P_1SS23GT	0	-1,046.70	-808.66	
21	CHI-MIR-194	0	-39,509.19	-44,671.03	
22	CHI-MIR-34C-5P	0	-61.30	-73.78	
23	PC-3P-37101_116	0	-19.28	-21.60	
24	PC-3P-3191_2100	0	-74.51	-63.31	
Gene Table for Profile 2 (0.0, -1.0,0.0)					0.982
1	CHI-MIR-211	0	-57.63	18.19	
2	CHI-MIR-214-3P_L-1R+3	0	-11,246.66	4,898.66	
3	CHI-MIR-214-5P	0	-102.99	-4.06	
4	CHI-MIR-329B-3P_R+1	0	-549.76	-247.17	
5	CHI-MIR-329A-3P_R+1_1SS19TC	0	-549.76	-247.17	
Gene Table for Profile 3 (0.0,0.0, -1.0)					0.805
1	CHI-MIR-98-5P	0	112.63	-284.83	
2	CHI-MIR-22-3P_R+3	0	2,895.81	-3,977.78	
3	CHI-MIR-494	0	1,290.21	-1,402.23	
4	CHI-MIR-340-5P	0	56.26	-95.29	
5	CHI-MIR-3959-5P	0	6.41	-202.94	
Gene Table for Profile 4 (0.0,0.0,1.0)					0.999
1	CHI-MIR-320-3P	0	5,101.08	27,566.81	
2	CHI-MIR-129-3P	0	-411.06	1,765.55	
3	CHI-MIR-150	0	1,459.83	7,589.36	
4	CHI-MIR-99A-3P	0	-53.36	110.12	
5	PC-3P-89392_15	0	0	3.92	
6	PC-5P-27762_176	0	-1.03	6.01	
Gene Table for Profile 5 (0.0,1.0,0.0)					0.794
1	CHI-MIR-186-5P_R+1	0	10,047.12	2,572.34	
2	CHI-MIR-146A	0	883.38	83.51	
3	CHI-MIR-146B-5P	0	1,726.04	63.26	
4	CHI-MIR-374B-3P	0	3.42	-1.24	
5	CHI-MIR-145-3P_L+2R-1	0	2,032.56	449.18	
6	CHI-MIR-33B-3P	0	10.7	-5.88	
7	CHI-MIR-1	0	1,889.91	106.05	
8	CHI-MIR-143-5P	0	2,936.31	-307.62	

9	CHI-MIR-30C-3P_R+1	0	50.96	11.68	
10	CHI-MIR-146B-3P	0	122.14	13.16	
11	PC-5P-62000_42	0	8.21	0	
12	PC-5P-18419_285	0	33.04	-9.76	
13	PC-3P-80363_21	0	4.18	0	
14	PC-3P-43684_88	0	6.85	-0.12	
15	PC-5P-3628_1784	0	161.3	37.74	
Gene Table for Profile 6 (0.0,1.0,1.0)					0.503
1	CHI-MIR-145-5P	0	380,983.06	431,889.00	
2	CHI-MIR-29B-5P	0	121.01	155.44	
3	CHI-MIR-222-3P_R+1	0	7,280.04	5,898.80	
4	CHI-MIR-423-5P	0	15,657.20	13,168.87	
5	CHI-MIR-181B-5P_R+1	0	5,541.94	6,290.89	
6	CHI-MIR-195-3P_R+1	0	288.78	194.9	
7	CHI-MIR-342-5P	0	88.3	75.06	
Gene Table for Profile 7 (0.0,1.0,2.0)					0.024
1	CHI-MIR-29C-5P_1SS23AT	0	162.94	239.2	
2	CHI-MIR-30F-5P_R+1	0	7,137.94	13,356.26	
3	CHI-LET-7B-5P	0	42,780.02	65,297.70	
4	CHI-MIR-92A-5P	0	15.46	25.16	
5	CHI-MIR-342-3P_R+2	0	13,790.70	22,293.55	
6	CHI-MIR-29A-3P_R+1	0	2,738.35	4,121.32	
7	CHI-MIR-671-5P	0	19.24	35.54	
8	CHI-MIR-425-5P_L-1R+2	0	354.58	625.13	
9	CHI-MIR-193B-5P	0	36.21	80.45	
10	CHI-MIR-491-5P_R+1	0	62.6	175.79	
11	CHI-MIR-23A_R+1	0	16,622.14	27,387.08	
12	PC-3P-20760_249	0	8.06	17.26	

Table S5: The identified DEmiRNAs in the goat colon.

miR_name	miR_seq	D10_1 (norm)	D10_2 (norm)	D10_3 (norm)	D10_4 (norm)	D10_5 (norm)	D25_1 (norm)	D25_2 (norm)	D25_3 (norm)	D25_4 (norm)	D25_5 (norm)	D31_1 (norm)	D31_2 (norm)	D31_3 (norm)	D31_4 (norm)	D31_5 (norm)
chi-miR-1185-3p	ATATACAGAGGGAGACTCTTAT	81.65648211	84.74874639	58.19111424	46.47866561	65.65286255	104.8024656	24.26300884	72.19982339	44.7115274	24.72095152	15.78211941	18.87339635	18.76543041	35.06738123	67.49276279
chi-miR-1197-3p	TAGGACACATGGTCTACTTCT	68.21933949	63.789379	63.1086732	68.01755943	84.2840803	51.4484831	62.5730228	47.3811341	49.41800397	39.55352244	25.74977378	31.14110398	43.16048995	25.42385139	39.2995834
chi-miR-124a	TAAGGCACGCGGTGAATGC	566.427243	780.9642544	885.9802042	1122.289731	868.5696276	412.5406145	291.1561061	219.6077961	392.9907934	442.0106133	528.2856814	619.9910701	437.2345286	571.598314	619.3956079
chi-miR-1271-3p	AGTGCCTGCTATGTGCCAGG	18.60527441	16.40298317	27.86616738	34.00877972	29.2776279	8.574747183	0	9.024977923	7.059714852	6.921866427	5.814465047	17.92972653	0	12.27358343	16.23243662
chi-miR-145-5p	GTCCAGTTTTCCCAGGAATCCCT	413928.0777	273737.5396	305373.8541	259564.0758	158024.6658	793964.4242	638968.8918	541464.8317	631723.2875	709422.0901	752564.5498	708834.7524	619420.5864	683159.9272	806093.4048
chi-miR-146a	TGAGAACTGAATTCCATAGGTT	575.7298802	414.6309635	298.3319097	253.9322219	347.7827314	1424.360782	1678.617112	2107.332345	423.5828911	673.3987195	539.0839737	334.0591154	394.0740387	307.7162703	733.0226642
chi-miR-150	TCTCCCAACCCTTGTACCAGTG	7238.48537	7862.496601	10018.70677	5326.908529	3185.051035	7577.218261	4807.906752	8794.088905	8113.965603	11637.63514	8922.711934	9453.684232	18551.50451	17071.67787	17578.87452

chi-miR-223-3p	TGTCAGTTTGTCAAATACCCCA	275.978237	1420.680598	475.3640319	671.1065864	1208.367551	5258.225523	247.7380903	7662.958338	1151.91014	2003.385912	2901.418059	451.0741728	2071.703518	2537.125032	3501.934616
chi-miR-26a-3p	CCTATTCTCGGTTACTTGCACG	7.235384491	6.3789379	4.097965792	10.20263391	4.436004227	4.763748435	7.662002792	4.512488962	8.236333994	0	4.153189319	4.718349088	0	0	4.271693847
chi-miR-29a-5p	ACTGATTTCTTTTGGTGTTCAGAGT	6.201758135	6.3789379	4.91755895	0	11.53361099	30.48798999	0	26.32285228	14.1194297	0	47.34635824	12.26770763	9.382715207	28.05390498	28.19317939
chi-miR-29b-3p	CTAGCACCATTTGAAATCAGTG	27.90791161	15.49170633	31.96413318	5.668129953	23.95442282	92.41671964	7.662002792	72.19982339	18.82590627	62.29679784	45.68508251	57.56385887	35.65431779	35.94406576	69.20144033
chi-miR-29b-5p	CTGGTTTCACATGGTGGCTTAGA	185.0191177	143.9817412	154.0835138	113.3625991	123.3209175	297.2579024	201.7660735	285.7909676	223.557637	316.4281795	299.8602689	433.1444463	283.3579992	252.4851448	228.1084515
chi-miR-29c-5p	TGACCGATTTCTCCTGGTGTTCT	231.53	232.38	188.51	190.45	147.28	476.37	257.95	311.36	382.40	376.75	430.27	523.74	365.93	431.33	434.86
chi-miR-323b	CACAATACACGGTCGGCCTCT	1368.521295	1368.737818	1870.311587	1953.237582	1209.254752	774.5854956	1236.13645	1116.088936	829.5164951	848.4230563	575.6320397	529.3987676	793.7777065	1118.649461	788.5546842
chi-miR-374b-3p	CTTATCAGGTTGTATTATCATT	6.201758135	0	0	0	0	5.716498122	0	6.768733442	5.88309571	4.944190305	0	0	0	0	0

chi-miR-376a	GTAGATTCTCCTTCTATGAGT	119.9006573	91.12768429	70.48501162	46.47866561	39.92403804	94.32221902	0	33.84366721	54.12448053	23.73211346	27.41104951	19.81706617	28.14814562	32.43732763	47.84297109
chi-miR-380-5p	ATGGTTGACCACAGAACATGCG	7.235384491	8.201491586	6.556745267	0	5.323205072	4.763748435	0	10.52914091	0	0	0	0	0	4.383422653	0
chi-miR-410-3p	AATATAACACAGATGGCCTGT	2767.017755	1924.616692	2273.551421	2535.921341	1837.392951	2004.585342	1154.408421	1331.936325	1354.288632	1165.840074	928.6531318	870.0635718	1352.987533	1084.458764	1398.552566
chi-miR-412-3p	CTTCACCTGGTCCACTAGCT	20.67252712	19.1368137	54.09314845	31.74152773	26.61602536	12.38574593	6.385002327	8.27289643	0	22.7432754	0	0	15.01234433	21.04042874	8.543387695
chi-miR-483	TCACTCCTCTCCTCCCGTCTTCT	262.5410944	375.4460593	375.3736665	637.0978067	226.2362156	263.9116633	90.66703304	217.3515516	220.0277796	233.3657824	313.1504747	208.5510297	501.0369921	621.5693322	524.5640045
chi-miR-493-5p	TTGTACATGGTAGGCTTTCATT	4999.650683	7253.76367	6637.88499	4949.411075	4818.387791	2541.936165	3025.214102	3008.325974	3475.732946	1959.877037	2622.323736	3930.38479	3009.975038	2527.481502	2423.759089
chi-miR-496-5p	GAGTCGGGTACTCGAATGGA	0	7.290214743	14.75267685	5.668129953	10.64641014	0	0	0	0	0	0	0	0	0	8.543387695
chi-miR-544-5p	TCTTGTTAAAAGGCAGATTCT	36.17692245	6.3789379	18.85064264	15.87076387	26.61602536	27.62974092	26.81700977	21.05828182	20.00252541	12.85489479	7.475740775	6.605688723	0	19.28705967	16.23243662
chi-miR-592	TTGTGTCAATATGCGATGATGT	28.94153796	19.1368137	18.85064264	0	23.06722198	0	0	0	0	0	0	5.662018905	0	7.013476245	0

chi-miR-656	AATATTATACAGTCAACCTCT	24.80703254	11.84659896	5.737152109	12.4698859	15.08241437	18.10224405	0	6.768733442	12.94281056	6.921866427	8.306378639	0	0	4.383422653	6.834710156
chi-miR-758	TTTGTGACCTGGTCCACTAAC	981.945038	677.0786943	954.8260295	919.3706783	981.2441349	759.3415006	759.8152769	588.8798095	598.8991433	439.0440991	387.0772446	453.9051822	463.5061312	412.9184139	469.8863232
PC-3p-34697_129	TCACGGCCTCTCTGCCCCCAGT	0	9.112768429	0	0	6.210405917	11.43299624	17.87800651	12.0333039	0	12.85489479	18.27403301	5.662018905	15.01234433	9.643529837	8.543387695
PC-3p-37101_116	GCGTCTCACTGAGCAGCATGT	28.94153796	25.5157516	60.64989372	34.00877972	33.71363212	23.81874218	0	9.024977923	25.88562112	27.68746571	13.29020582	7.54935854	16.88888737	14.90363702	22.21280801
PC-3p-37151_116	TGGTGCTCCCTGGAGCTGAGCA	9.302637203	15.49170633	9.835117901	10.20263391	19.5184186	9.52749687	6.38502327	12.0333039	0	10.87721867	12.45956796	4.718349088	0	0	6.834710156
PC-3p-37745_113	TGTACTCTGGGACTCGGGTGTCT	13.9539558	19.59245212	6.556745267	12.4698859	15.96961522	18.5786189	84.92053094	26.69889302	5.88309571	28.18188474	6.229783979	6.605688723	13.13580129	4.383422653	0
PC-3p-43684_88	AAAACCCGCATGAACTTTTTTGG	7.235384491	6.3789379	4.097965792	0	0	10.48024656	19.15500698	6.016651949	9.412953136	6.921866427	4.983827183	0	0	7.013476245	5.126032617

PC-5p-18419_285	AGTTGAGGCTCCGAGCTTTGGT	17.57164805	48.29767267	22.12901528	19.27164184	29.2776279	40.96823654	103.4370377	61.67068247	54.12448053	41.53119856	14.12084369	22.64807562	15.01234433	35.94406576	0
PC-5p-24127_209	CTATGGTTTTCTGAGGAT	13.43714263	29.16085897	24.58779475	32.87515373	50.57044818	16.19674468	12.77000465	15.79371137	0	19.77676122	21.59658446	13.21137745	9.382715207	29.80727404	23.92148555
PC-5p-3628_1784	GAGGGTTTGGGTTTGGTCGTGGGA	200.523513	68.34576322	116.3822285	97.49183519	86.94568284	235.3291727	287.3251047	459.5217926	108.2489611	285.7741996	115.4586631	66.05688723	91.95060903	255.1151984	229.817129
PC-5p-43003_91	TGGTGGAACCGAGTCCTGGGT	5.168131779	6.3789379	7.376338425	10.20263391	8.872008453	5.716498122	0	10.52914091	10.58957228	7.910704488	0	0	0	6.136791714	5.980371386
PC-5p-48419_72	CGCAGAGGCGGGAGGTATGACCT	5.168131779	9.112768429	13.11349053	17.00438986	11.53361099	8.574747183	28.09401024	3.760407468	8.236333994	6.921866427	7.475740775	0	0	7.013476245	5.126032617
PC-5p-49834_68	AACCCGGTCCGCCTCCCCCCG	0	0	4.097965792	0	0	0	6.385002327	29.33117825	0	11.86605673	7.475740775	4.718349088	15.01234433	23.67048233	34.17355078
PC-5p-53455_59	AAGAGGGTAACTATACAGTGAA	0	0	2.868576054	0	0	6.192872966	0	5.264570455	3.529857426	2.966514183	5.399146115	0	0	2.191711327	0
PC-5p-62000_42	AGCTGGAGACTACTGAAGTGAC	0	0	0	0	0	0	7.662002792	13.53746688	12.94281056	6.921866427	0	0	0	0	0

Table S6: The enriched KEGG pathway of differentially expressed miRNAs in goat colon from D10 to D31

Pathway Id	Pathway description	S gene number	TS gene number	B gene number	TB gene number	*P*-value of Fisher's Exact Test
ko04010	MAPK signaling pathway	1,254	18,333	1,719	29,837	3.44E-25
ko04931	Insulin resistance	470	18,333	605	29,837	5.46E-18
ko04910	Insulin signaling pathway	439	18,333	566	29,837	1.13E-16
ko04810	Regulation of actin cytoskeleton	1,074	18,333	1,511	29,837	5.51E-16
ko05166	HTLV-I infection	533	18,333	725	29,837	2.76E-12
ko04510	Focal adhesion	1,074	18,333	1,542	29,837	3.14E-12
ko04360	Axon guidance	516	18,333	702	29,837	6.46E-12
ko04390	Hippo signaling pathway	403	18,333	536	29,837	7.53E-12
ko05100	Bacterial invasion of epithelial cells	229	18,333	286	29,837	7.92E-12
ko05205	Proteoglycans in cancer	524	18,333	717	29,837	2.12E-11
ko04012	ErbB signaling pathway	669	18,333	934	29,837	2.18E-11
ko04514	Cell adhesion molecules	508	18,333	694	29,837	2.83E-11
ko04310	Wnt signaling pathway	505	18,333	690	29,837	3.36E-11
ko05200	Pathways in cancer	834	18,333	1,188	29,837	7.66E-11
ko05202	Transcriptional misregulation in cancers	613	18,333	855	29,837	1.16E-10
ko04071	Sphingolipid signaling pathway	273	18,333	356	29,837	5.59E-10
ko04068	FoxO signaling pathway	454	18,333	625	29,837	1.81E-09
ko00562	Inositol phosphate metabolism	202	18,333	258	29,837	4.77E-09
ko04320	Dorso-ventral axis formation	263	18,333	346	29,837	5.25E-09
ko04520	Adherens junction	445	18,333	615	29,837	6.04E-09
ko04666	Fc gamma R-mediated phagocytosis	238	18,333	311	29,837	9.65E-09
ko00310	Lysine degradation	228	18,333	297	29,837	1.21E-08
ko03420	Nucleotide excision repair	290	18,333	389	29,837	2.66E-08
ko04550	Signaling pathways regulating pluripotency of stem cells	424	18,333	590	29,837	5.29E-08
ko05217	Basal cell carcinoma	134	18,333	167	29,837	1.30E-07
ko04660	T cell receptor signaling pathway	218	18,333	290	29,837	4.66E-07
ko04070	Phosphatidylinositol signaling system	298	18,333	412	29,837	1.94E-06
ko04380	Osteoclast differentiation	267	18,333	366	29,837	2.11E-06

ko04933	AGE-RAGE signaling pathway in diabetic complications	277	18,333	382	29,837	3.13E-06
ko05213	Endometrial cancer	131	18,333	169	29,837	6.03E-06
ko04152	AMPK signaling pathway	265	18,333	366	29,837	6.14E-06
ko04062	Chemokine signaling pathway	324	18,333	455	29,837	7.03E-06
ko04144	Endocytosis	1,033	18,333	1,550	29,837	7.43E-06
ko05220	Chronic myeloid leukemia	167	18,333	222	29,837	9.11E-06
ko04664	Fc epsilon RI signaling pathway	149	18,333	196	29,837	1.01E-05
ko04022	cGMP - PKG signaling pathway	347	18,333	492	29,837	1.35E-05
ko04014	Ras signaling pathway	504	18,333	733	29,837	1.78E-05
ko04370	VEGF signaling pathway	185	18,333	250	29,837	1.79E-05
ko04072	Phospholipase D signaling pathway	307	18,333	433	29,837	2.12E-05
ko04150	mTOR signaling pathway	217	18,333	298	29,837	2.21E-05
ko04120	Ubiquitin mediated proteolysis	588	18,333	864	29,837	2.43E-05
ko04151	PI3K-Akt signaling pathway	618	18,333	911	29,837	2.72E-05
ko04611	Platelet activation	252	18,333	351	29,837	2.78E-05
ko05210	Colorectal cancer	156	18,333	209	29,837	3.65E-05
ko05215	Prostate cancer	209	18,333	289	29,837	6.30E-05
ko04214	Apoptosis - fly	64	18,333	78	29,837	6.98E-05
ko05206	MicroRNAs in cancer	419	18,333	610	29,837	1.00E-04
ko04020	Calcium signaling pathway	466	18,333	685	29,837	1.70E-04
ko04211	Longevity regulating pathway - mammal	191	18,333	265	29,837	1.73E-04
ko04723	Retrograde endocannabinoid signaling	203	18,333	284	29,837	2.38E-04
ko04015	Rap1 signaling pathway	492	18,333	727	29,837	2.40E-04
ko05218	Melanoma	152	18,333	208	29,837	2.67E-04
ko05032	Morphine addiction	166	18,333	229	29,837	2.81E-04
ko04916	Melanogenesis	254	18,333	362	29,837	3.04E-04
ko04662	B cell receptor signaling pathway	165	18,333	228	29,837	3.34E-04
ko04921	Oxytocin signaling pathway	336	18,333	488	29,837	3.55E-04
ko05222	Small cell lung cancer	189	18,333	265	29,837	4.61E-04
ko05014	Amyotrophic lateral sclerosis	107	18,333	143	29,837	4.94E-04
ko05212	Pancreatic cancer	134	18,333	183	29,837	5.20E-04
ko04728	Dopaminergic synapse	278	18,333	403	29,837	9.10E-04

ko00532	Glycosaminoglycan biosynthesis - chondroitin sulfate / dermatan sulfate	50	18,333	62	29,837	9.51E-04
ko00460	Cyanoamino acid metabolism	36	18,333	43	29,837	1.37E-03
ko05216	Thyroid cancer	101	18,333	137	29,837	1.66E-03
ko00230	Purine metabolism	359	18,333	531	29,837	1.70E-03
ko04110	Cell cycle	395	18,333	588	29,837	2.07E-03
ko04710	Circadian rhythm	80	18,333	107	29,837	2.51E-03
ko05120	Epithelial cell signaling in Helicobacter pylori infection	16	18,333	17	29,837	2.95E-03
ko05231	Choline metabolism in cancer	210	18,333	304	29,837	3.23E-03
ko00603	Glycosphingolipid biosynthesis - globo series	43	18,333	54	29,837	3.42E-03
ko04350	TGF-beta signaling pathway	263	18,333	386	29,837	3.53E-03
ko05211	Renal cell carcinoma	123	18,333	172	29,837	3.60E-03
ko05219	Bladder cancer	79	18,333	107	29,837	4.75E-03
ko05223	Non-small cell lung cancer	127	18,333	179	29,837	4.88E-03
ko05230	Central carbon metabolism in cancer	146	18,333	208	29,837	5.14E-03
ko00232	Caffeine metabolism	34	18,333	42	29,837	5.58E-03
ko04261	Adrenergic signaling in cardiomyocytes	407	18,333	613	29,837	5.85E-03
ko05221	Acute myeloid leukemia	143	18,333	204	29,837	6.02E-03
ko04914	Progesterone-mediated oocyte maturation	227	18,333	333	29,837	6.13E-03
ko00920	Sulfur metabolism	36	18,333	45	29,837	6.31E-03
ko04919	Thyroid hormone signaling pathway	243	18,333	358	29,837	6.47E-03
ko00750	Vitamin B6 metabolism	14	18,333	15	29,837	6.98E-03
ko05412	Arrhythmogenic right ventricular cardiomyopathy	227	18,333	334	29,837	7.56E-03
ko04960	Aldosterone-regulated sodium reabsorption	101	18,333	142	29,837	1.00E-02
ko04064	NF-kappa B signaling pathway	191	18,333	280	29,837	1.07E-02
ko05214	Glioma	146	18,333	211	29,837	1.14E-02
ko00730	Thiamine metabolism	9	18,333	9	29,837	1.25E-02
ko03018	RNA degradation	262	18,333	391	29,837	1.25E-02
ko00430	Taurine and hypotaurine metabolism	21	18,333	25	29,837	1.37E-02
ko00062	Fatty acid elongation	53	18,333	72	29,837	2.07E-02
ko05132	Salmonella infection	210	18,333	313	29,837	2.16E-02
ko04725	Cholinergic synapse	206	18,333	307	29,837	2.26E-02
ko04530	Tight junction	648	18,333	1,005	29,837	2.36E-02

ko00270	Cysteine and methionine metabolism	99	18,333	142	29,837	2.46E-02
ko00534	Glycosaminoglycan biosynthesis - heparan sulfate / heparin	52	18,333	71	29,837	2.52E-02
ko04912	GnRH signaling pathway	234	18,333	352	29,837	2.81E-02
ko00670	One carbon pool by folate	39	18,333	52	29,837	2.84E-02
ko05142	Chagas disease	185	18,333	276	29,837	3.10E-02
ko04727	GABAergic synapse	169	18,333	252	29,837	3.69E-02
ko04721	Synaptic vesicle cycle	142	18,333	210	29,837	3.69E-02
ko04668	TNF signaling pathway	211	18,333	318	29,837	3.92E-02
ko04141	Protein processing in endoplasmic reticulum	416	18,333	642	29,837	4.18E-02
ko04130	SNARE interactions in vesicular transport	51	18,333	71	29,837	4.46E-02
ko05033	Nicotine addiction	74	18,333	106	29,837	4.55E-02

Table S7: The enriched KEGG pathway of highly expressed miRNAs in goat colon from D10 to D31

Pathway Id	Pathway description	S gene number	TS gene number	B gene number	TB gene number	*P*-value of Fisher's Exact Test
ko04310	Wnt signaling pathway	609	21,529	690	29,837	3.12E-25
ko04514	Cell adhesion molecules	606	21,529	694	29,837	2.03E-22
ko04010	MAPK signaling pathway	1,392	21,529	1,719	29,837	1.91E-18
ko04520	Adherens junction	531	21,529	615	29,837	1.61E-17
ko04530	Tight junction	824	21,529	1,005	29,837	1.07E-13
ko04360	Axon guidance	582	21,529	702	29,837	1.09E-11
ko04931	Insulin resistance	506	21,529	605	29,837	1.48E-11
ko05200	Pathways in cancer	955	21,529	1,188	29,837	1.52E-11
ko05166	HTLV-I infection	599	21,529	725	29,837	1.87E-11
ko04320	Dorso-ventral axis formation	301	21,529	346	29,837	2.21E-11
ko04390	Hippo signaling pathway	449	21,529	536	29,837	1.29E-10
ko04070	Phosphatidylinositol signaling system	350	21,529	412	29,837	4.47E-10
ko04510	Focal adhesion	1,215	21,529	1,542	29,837	5.43E-10
ko05212	Pancreatic cancer	165	21,529	183	29,837	1.83E-09
ko04120	Ubiquitin mediated proteolysis	695	21,529	864	29,837	7.51E-09
ko04910	Insulin signaling pathway	466	21,529	566	29,837	8.24E-09
ko04810	Regulation of actin cytoskeleton	1,184	21,529	1,511	29,837	9.42E-09
ko05205	Proteoglycans in cancer	582	21,529	717	29,837	9.83E-09
ko03420	Nucleotide excision repair	327	21,529	389	29,837	1.91E-08
ko00562	Inositol phosphate metabolism	223	21,529	258	29,837	2.97E-08
ko04014	Ras signaling pathway	592	21,529	733	29,837	3.16E-08
ko04068	FoxO signaling pathway	509	21,529	625	29,837	3.48E-08
ko04660	T cell receptor signaling pathway	248	21,529	290	29,837	4.31E-08
ko05215	Prostate cancer	247	21,529	289	29,837	5.13E-08
ko05220	Chronic myeloid leukemia	193	21,529	222	29,837	9.49E-08
ko04720	Long-term potentiation	200	21,529	231	29,837	1.14E-07
ko05211	Renal cell carcinoma	152	21,529	172	29,837	2.10E-07
ko04921	Oxytocin signaling pathway	400	21,529	488	29,837	2.49E-07
ko04012	ErbB signaling pathway	739	21,529	934	29,837	4.32E-07
ko05206	MicroRNAs in cancer	492	21,529	610	29,837	6.15E-07
ko04114	Oocyte meiosis	355	21,529	434	29,837	1.73E-06

ko04916	Melanogenesis	299	21,529	362	29,837	2.13E-06
ko04330	Notch signaling pathway	248	21,529	297	29,837	2.88E-06
ko04350	TGF-beta signaling pathway	317	21,529	386	29,837	3.02E-06
ko00601	Glycosphingolipid biosynthesis - lacto and neolacto series	87	21,529	96	29,837	7.96E-06
ko04728	Dopaminergic synapse	327	21,529	403	29,837	1.71E-05
ko05214	Glioma	177	21,529	211	29,837	4.27E-05
ko00533	Glycosaminoglycan biosynthesis - keratan sulfate	50	21,529	53	29,837	4.79E-05
ko04550	Signaling pathways regulating pluripotency of stem cells	467	21,529	590	29,837	5.10E-05
ko04662	B cell receptor signaling pathway	190	21,529	228	29,837	5.15E-05
ko05217	Basal cell carcinoma	142	21,529	167	29,837	6.04E-05
ko04110	Cell cycle	465	21,529	588	29,837	6.21E-05
ko04919	Thyroid hormone signaling pathway	290	21,529	358	29,837	6.37E-05
ko04072	Phospholipase D signaling pathway	347	21,529	433	29,837	7.38E-05
ko04933	AGE-RAGE signaling pathway in diabetic complications	308	21,529	382	29,837	7.69E-05
ko05219	Bladder cancer	94	21,529	107	29,837	7.71E-05
ko04727	GABAergic synapse	208	21,529	252	29,837	7.84E-05
ko04724	Glutamatergic synapse	257	21,529	316	29,837	9.65E-05
ko04713	Circadian entrainment	249	21,529	306	29,837	1.15E-04
ko04723	Retrograde endocannabinoid signaling	232	21,529	284	29,837	1.20E-04
ko00062	Fatty acid elongation	65	21,529	72	29,837	1.56E-04
ko04144	Endocytosis	1,180	21,529	1,550	29,837	1.56E-04
ko00232	Caffeine metabolism	40	21,529	42	29,837	1.61E-04
ko04925	Aldosterone synthesis and secretion	226	21,529	277	29,837	1.70E-04
ko04380	Osteoclast differentiation	294	21,529	366	29,837	1.84E-04
ko00310	Lysine degradation	241	21,529	297	29,837	2.07E-04
ko04015	Rap1 signaling pathway	566	21,529	727	29,837	2.32E-04
ko05412	Arrhythmogenic right ventricular cardiomyopathy	269	21,529	334	29,837	2.44E-04
ko04668	TNF signaling pathway	256	21,529	318	29,837	3.59E-04
ko00920	Sulfur metabolism	42	21,529	45	29,837	4.08E-04
ko05210	Colorectal cancer	172	21,529	209	29,837	4.24E-04
ko05031	Amphetamine addiction	182	21,529	222	29,837	4.34E-04
ko04725	Cholinergic synapse	247	21,529	307	29,837	4.83E-04
ko05218	Melanoma	171	21,529	208	29,837	4.87E-04
ko05230	Central carbon metabolism in cancer	171	21,529	208	29,837	4.87E-04

ko04022	cGMP - PKG signaling pathway	386	21,529	492	29,837	7.78E-04
ko00770	Pantothenate and CoA biosynthesis	49	21,529	54	29,837	7.87E-04
ko05161	Hepatitis B	287	21,529	361	29,837	7.93E-04
ko05221	Acute myeloid leukemia	167	21,529	204	29,837	8.41E-04
ko04141	Protein processing in endoplasmic reticulum	498	21,529	642	29,837	9.40E-04
ko04664	Fc epsilon RI signaling pathway	160	21,529	196	29,837	1.37E-03
ko00532	Glycosaminoglycan biosynthesis - chondroitin sulfate / dermatan sulfate	55	21,529	62	29,837	1.46E-03
ko05130	Pathogenic Escherichia coli infection	37	21,529	40	29,837	1.49E-03
ko04146	Peroxisome	227	21,529	284	29,837	1.57E-03
ko00270	Cysteine and methionine metabolism	118	21,529	142	29,837	1.61E-03
ko04922	Glucagon signaling pathway	288	21,529	365	29,837	1.84E-03
ko05202	Transcriptional misregulation in cancers	654	21,529	855	29,837	2.03E-03
ko05223	Non-small cell lung cancer	146	21,529	179	29,837	2.32E-03
ko00460	Cyanoamino acid metabolism	39	21,529	43	29,837	2.88E-03
ko04214	Apoptosis - fly	67	21,529	78	29,837	3.16E-03
ko04211	Longevity regulating pathway - mammal	211	21,529	265	29,837	3.20E-03
ko05120	Epithelial cell signaling in Helicobacter pylori infection	17	21,529	17	29,837	3.89E-03
ko00514	Other types of O-glycan biosynthesis	91	21,529	109	29,837	4.05E-03
ko04020	Calcium signaling pathway	525	21,529	685	29,837	4.07E-03
ko00564	Glycerophospholipid metabolism	261	21,529	332	29,837	4.18E-03
ko04071	Sphingolipid signaling pathway	279	21,529	356	29,837	4.30E-03
ko04611	Platelet activation	275	21,529	351	29,837	4.69E-03
ko04512	ECM-receptor interaction	344	21,529	443	29,837	4.74E-03
ko05213	Endometrial cancer	137	21,529	169	29,837	4.85E-03
ko05014	Amyotrophic lateral sclerosis	117	21,529	143	29,837	4.95E-03
ko05169	Epstein-Barr virus infection	423	21,529	549	29,837	5.01E-03
ko05160	Hepatitis C	250	21,529	319	29,837	6.61E-03
ko04540	Gap junction	224	21,529	285	29,837	7.71E-03
ko04914	Progesterone-mediated oocyte maturation	260	21,529	333	29,837	7.98E-03
ko05231	Choline metabolism in cancer	238	21,529	304	29,837	8.62E-03
ko04912	GnRH signaling pathway	274	21,529	352	29,837	8.71E-03
ko01040	Biosynthesis of unsaturated fatty acids	61	21,529	72	29,837	9.25E-03
ko05145	Toxoplasmosis	231	21,529	295	29,837	9.40E-03

ko05032	Morphine addiction	181	21,529	229	29,837	1.04E-02
ko04024	cAMP signaling pathway	476	21,529	624	29,837	1.05E-02
ko00534	Glycosaminoglycan biosynthesis - heparan sulfate / heparin	60	21,529	71	29,837	1.10E-02
ko04130	SNARE interactions in vesicular transport	60	21,529	71	29,837	1.10E-02
ko00100	Steroid biosynthesis	66	21,529	79	29,837	1.32E-02
ko03013	RNA transport	552	21,529	728	29,837	1.33E-02
ko00512	Mucin type O-glycan biosynthesis	68	21,529	82	29,837	1.66E-02
ko05020	Prion diseases	71	21,529	86	29,837	1.76E-02
ko00410	beta-Alanine metabolism	67	21,529	81	29,837	1.93E-02
ko04710	Circadian rhythm	87	21,529	107	29,837	1.94E-02
ko04115	p53 signaling pathway	227	21,529	293	29,837	2.24E-02
ko04911	Insulin secretion	246	21,529	319	29,837	2.55E-02
ko00510	N-Glycan biosynthesis	126	21,529	159	29,837	2.55E-02
ko04340	Hedgehog signaling pathway	177	21,529	227	29,837	2.74E-02
ko04062	Chemokine signaling pathway	346	21,529	455	29,837	3.36E-02
ko03015	mRNA surveillance pathway	347	21,529	457	29,837	3.76E-02
ko04013	MAPK signaling pathway - fly	10	21,529	10	29,837	3.82E-02
ko05203	Viral carcinogenesis	481	21,529	639	29,837	4.03E-02
ko04730	Long-term depression	145	21,529	186	29,837	4.33E-02
ko00120	Primary bile acid biosynthesis	41	21,529	49	29,837	4.53E-02
ko04971	Gastric acid secretion	174	21,529	225	29,837	4.59E-02
ko04215	Apoptosis - multiple species	105	21,529	133	29,837	4.62E-02
ko00450	Selenocompound metabolism	54	21,529	66	29,837	4.88E-02
